# Vessel-Guided Mesohepatectomy for Liver Partition and Staged Major Parenchyma-Sparing Hepatectomies with Super-Selective Portal Vein Embolization or Enhanced ALPPS to Achieve R0 Resection for Colorectal Liver Metastases at the Hepatocaval Confluence

**DOI:** 10.3390/cancers15194683

**Published:** 2023-09-22

**Authors:** Lucio Urbani, Nicolò Roffi, Roberto Moretto, Stefano Signori, Riccardo Balestri, Elisabetta Rossi, Piero Colombatto, Gabriella Licitra, Chiara Leoni, Rita Martinelli, Daniele Anacleto Meiattini, Emidio Bonistalli, Beatrice Borelli, Carlotta Antoniotti, Gianluca Masi, Daniele Rossini, Piero Boraschi, Francescamaria Donati, Maria Clotilde Della Pina, Alessandro Lunardi, Francesco Daviddi, Laura Crocetti, Michele Tonerini, Roberto Gigoni, Francesca Quilici, Raffaele Gaeta, Francesca Turco, Adriana Paolicchi, Duccio Volterrani, Vincenzo Nardini, Piero Buccianti, Francesco Forfori, Marco Puccini, Chiara Cremolini

**Affiliations:** 1 General Surgery Unit, Azienda Ospedaliero-Universitaria Pisana, 56124 Pisa, Italy; nicolo.roffi@gmail.com (N.R.); s.signori@ao-pisa.toscana.it (S.S.); r.balestri@ao-pisa.toscana.it (R.B.); elyrossi@hotmail.com (E.R.); f.turco3@studenti.unipi.it (F.T.); p.buccianti@ao-pisa.toscana.it (P.B.); marco.puccini@med.unipi.it (M.P.); 2 Oncology Unit, Azienda Ospedaliero-Universitaria Pisana, 56124 Pisa, Italy; robertomoretto8468@gmail.com (R.M.); b.borelli@gmail.com (B.B.); carlottantoniotti@gmail.com (C.A.); gianlucamasi72@gmail.com (G.M.); danielerossini87@gmail.com (D.R.); chiaracremolini@gmail.com (C.C.); 3 Hepatology Unit, Azienda Ospedaliero-Universitaria Pisana, 56124 Pisa, Italy; p.colombatto@ao-pisa.toscana.it; 4 Anaesthesiology and Intensive Care Unit, Azienda Ospedaliero-Universitaria Pisana, 56124 Pisa, Italy; gabriellalicitra@gmail.com (G.L.); chiara.leoni@me.com (C.L.); rita.martinelli@gmail.com (R.M.); daniele.meiattini@ao-pisa.toscana.it (D.A.M.); emidio.bonistalli@ao-pisa.toscana.it (E.B.); a.paolicchi@ao-pisa.toscana.it (A.P.); francescoforfori@gmail.com (F.F.); 5 Radiology Unit, Azienda Ospedaliero-Universitaria Pisana, 56124 Pisa, Italy; p.boraschi@gmail.com (P.B.); francescamaria69@gmail.com (F.D.); clotilde.dellapina@gmail.com (M.C.D.P.); alunardi@sirm.org (A.L.); francescodaviddi@gmail.com (F.D.); laura.crocetti@med.unipi.it (L.C.); m.tonerini@ao-pisa.toscana.it (M.T.); robertogigoni@virgilio.it (R.G.); 6 Pathology Unit, Azienda Ospedaliero-Universitaria Pisana, 56124 Pisa, Italy; francesca.quilici@ao-pisa.toscana.it (F.Q.); raffaele.gaeta@med.unipi.it (R.G.); v.nardini@ao-pisa.toscana.it (V.N.); 7 Nuclear Medicine Unit, Azienda Ospedaliero-Universitaria Pisana, 56124 Pisa, Italy; duccio.volterrani@med.unipi.it

**Keywords:** R0, liver resection, hepatic vein, liver metastasis, parenchyma sparing, hepatocaval confluence, ALPPS, TSH, PVE

## Abstract

**Simple Summary:**

Up to 80% of disease relapse within 2 years after resection of colorectal cancer liver metastases occurs in patients with high burden of disease. Increase in life expectancy with new chemotherapy protocols opens the new challenge of maintaining quality of life after disease relapse and of offering subsequent surgical options when feasible. First-order glissonean-pedicles division recurrence should be absolutely avoided since it may hamper the subsequent administration of systemic treatments and worsen patient’s quality of life due to biliary involvement. To this end, selected cases of metastases at the hepatocaval-confluence were treated with vessel-guided mesohepatectomy of segments 1 and 4 *en-bloc* with the middle hepatic vein. This minor liver resection induces a major hepatic scaffold modification, transforming the liver into a paired organ. This novelty led us to use for the first time liver augmentation techniques in a parenchyma-sparing context, observing a liver regeneration never described before. Based on our results, this surgical approach may represent a new option for patients affected by colorectal liver metastases selected in the frame of an experienced multidisciplinary environment including dedicated oncologists, anesthesiologists, radiologists, pathologists and surgeons.

**Abstract:**

*Background*. R0 minor parenchyma-sparing hepatectomy (PSH) is feasible for colorectal liver metastases (CRLM) in contact with hepatic veins (HV) at hepatocaval confluence since HV can be reconstructed, but in the case of contact with the first-order glissonean pedicle (GP), major hepatectomy is mandatory. To pursue an R0 parenchyma-sparing policy, we proposed vessel-guided mesohepatectomy for liver partition (MLP) and eventually combination with liver augmentation techniques for staged major PSH. *Methods*. We analyzed 15 consecutive vessel-guided MLPs for CRLM at the hepatocaval confluence. Patients had a median of 11 (range: 0–67) lesions with a median diameter of 3.5 cm (range: 0.0–8.0), bilateral in 73% of cases. *Results*. Grade IIIb or more complications occurred in 13%, median hospital stay was 14 (range: 6–62) days, 90-day mortality was 0%. After a median follow-up of 17.5 months, 1-year OS and RFS were 92% and 62%. In nine (64%) patients, MLP was combined with portal vein embolization (PVE) or ALPPS to perform staged R0 major PSH. Future liver remnant (FLR) volume increased from a median of 15% (range: 7–20%) up to 41% (range: 37–69%). Super-selective PVE was performed in three (33%) patients and enhanced ALPPS (e-ALPPS) in six (66%). In two e-ALPPS an intermediate stage of deportalized liver PSH was necessary to achieve adequate FLR volume. *Conclusions*. Vessel-guided MLP may transform the liver in a paired organ. In selected cases of multiple bilobar CRLM, to guarantee oncological radicality (R0), major PSH is feasible combining advanced surgical parenchyma sparing with liver augmentation techniques when FLR volume is insufficient.

## 1. Introduction

In recent years parenchyma-sparing techniques are progressively gaining higher relevance in the surgical treatment of complex cases of bilobar colorectal liver metastases (CRLM) [1]. Enhanced one-stage hepatectomy (e-OSH) was introduced to resect less than three adjacent liver segments, avoiding classical major hepatectomies and the need to increase future liver remnant (FLR) using two-stage hepatectomy, portal vein embolization (PVE) or portal vein ligation (PVL) associated to liver partition (ALPPS) [2,3,4,5,6]. In recent years, Torzilli et al. pushed the limits of e-OSH resecting more than three adjacent liver segments, introducing the new parenchyma-sparing vessel-guided major hepatectomy paradigm [7]. The cornerstone of e-OSH is “tumor detachment” from main intrahepatic vessels [8], furthermore, R1 resections, in the case of multiple CRLMs, are considered by surgeons and oncologists to have a modest negative prognostic value as compared with R0 resections [9].

Since the beginning of our experience we have integrated liver-sparing surgical techniques with vascular reconstructions [10,11], making it possible to pursue a parenchyma-sparing policy associated with R0 liver resection in selected cases [12]. Describing the new parenchyma-sparing hepatectomy (PSH) with double HV resection and direct reconstruction in the case of CRLM in contact with two HVs at the hepatocaval confluence, we observed, apparently differently from the detachment HV management [13], that HV involvement is not predictable prior to histological analysis as HV infiltration is possible regardless of the degree of contact between CRLM and the vessel circumference [12]. Taking into account this observation, even if the oncological outcome of the “detachment technique” is favorable in a large series of chemotherapy responsive diseases [8], we considered that vessel detachment may increase the chance of local recurrence. Thus, intraoperative management of HV detachment is different from that of glissonean pedicle (GP). When needed, tangential resection of the vessel is possible in the first situation [13], but not in the second when complete amputation is mandatory. Clinical impact of local recurrence is also different since HV can be resected with limited liver resections as shown [14], but GP requires major hepatectomies and may hamper the administration of systemic treatments while worsening the patient’s quality of life due to biliary tree and portal vein involvement. The worst location for a local recurrence is the first-order GP confluence. With the aim of reducing this risk, we reconsidered R0 resections for selected cases of CRLM between HVs and the origin of the first-order GP. We proposed the complete removal of liver parenchyma at the origin of both left and right GPs, performing a mesohepatectomy for liver partition. As a consequence, we started thinking of the liver as a paired organ [15,16], introducing new staged parenchyma-sparing strategies in the case of insufficient future liver remnant (FLR), integrating advanced parenchyma-sparing surgical techniques with liver augmentation techniques such as PVE and ALPPS to pursue an R0 policy with parenchyma-sparing major hepatectomies. At the first stage, the diseased liver is divided in two independent organs planning firstly the resection of CRLM in “one of the two livers”, and secondly the independent treatment of the liver with CRLM still in place. This second liver can be used to allow the regeneration of the FLR (the liver without CRLM) in the case of first-order GP resection, or it can be preserved with a vessel-guided hepatectomy after a super-selective PVE if one second-order GP can be saved. Here, we describe all consecutive cases of vessel-guided mesohepatectomy for liver partition (MLP) performed at our center to treat CRLM located at the hepatocaval confluence, analyzing all new staged parenchyma-sparing strategies introduced to treat multiple bilobar complex CRLM with R0 major parenchyma-sparing liver resections and insufficient FLR volume.

## 2. Patients and Methods

### 2.1. General Definitions

The terminology for liver anatomy and resections was based on the Brisbane classification [17]. Hepatic resections were considered major when at least 3 adjacent liver segments were removed. The hepatocaval confluence was defined as the last 4 cm tract of each HV prior to its confluence into the inferior vena cava (IVC) and its intraoperative management required the complete mobilization of the paracaval portion of S1 from IVC suturing and sectioning all accessory HVs. Response to preoperative chemotherapy was classified according to the Response Evaluation Criteria in Solid Tumors criteria v. 1.1 [18]. Postoperative complications were stratified according to Dindo–Clavien classification [19]. Major complications (grade IIIb–IV) and operative mortality (grade V) were defined when they occurred within one month from the date of PSH or during the hospital stay even when longer than one month. The width of resection margin was defined as the shortest microscopic distance from tumoral edge to transection line. Local tumor recurrence was defined as any cut-edge recurrence.

### 2.2. MLP (Mesohepatectomy for Liver Partition)

Complete separation of left and right liver is achieved performing a mesohepatectomy involving segments S4 and/or S5/S8 *en-bloc* with S1 or the paracaval portion only and/or the caudate processus. At the end of liver partition, two independent livers are obtained, with regular inflow and outflow and biliary drainage. The confluence between first-order GPs is always exposed completely (vessel-guided hepatectomy) and the middle hepatic vein (MHV) is usually resected.

### 2.3. SS-PVE (Super-Selective Portal Vein Embolization)

After MLP, the portal vein of the liver with CRLM in place is super-selectively embolized with the aim of preserving at least one second-order portal branch.

To catheterize each portal branch, a 5F reverse-curve hydrophilic coated catheter (GLIDECATH^®^ Hydrophilic Coated Catheter Sim2 and Cobra 2C, Terumo, Tokyo, Japan) was used. The embolization was performed using a mixture composed of iodized oil (LIPIODOL, Guerbet, Aulnay-sous-bois, France) and n-butyl-cyanoacrylate (Glubran 2, GEM, Viareggio, Italy) with a 7:1 ratio. The goal of PVE was to achieve occlusion of the selected segmental branches of tumor-bearing liver based on the pre-op plan. At the end of each procedure, a track embolization was performed by injecting a small amount of glue into the parenchymal space between the portal vein and the liver surface. All patients received anticoagulant therapy after PVE to reduce the risk of portal vein thrombosis.

### 2.4. PS-TSH with SS-PVE (Parenchyma-Sparing TWO-STAGE Hepatectomy with Super-Selective Portal Vein Embolization)

**Figure 1:**
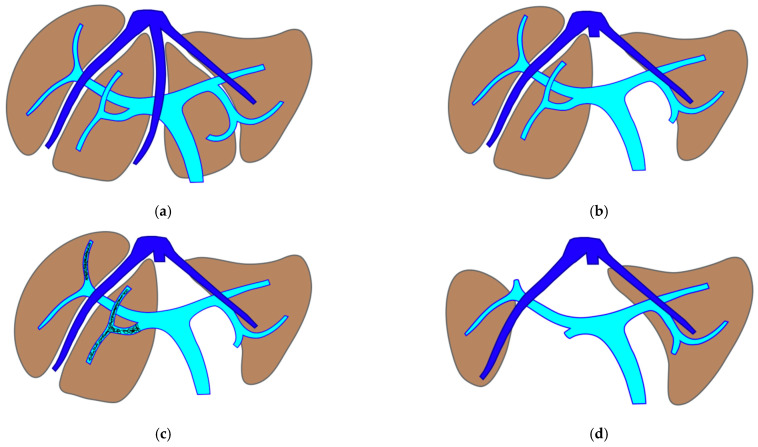
PS-TSH with SS-PVE. The parenchyma-sparing TWO-STAGE major hepatectomy with super-selective PVE is proposed when preservation of a second-order GP is planned and FLR volume is less than 40%. (**a**) Whole liver. (**b**) First stage: vessel-guided mesohepatectomy of S1/S4 *en-bloc* with MHV for liver partition (MLP). (**c**) Super-selective PVE of the anterior portal branch for S5/S8 and of the portal branch for S7, preserving the portal branch for S6. (**d**) Second stage: vessel-guided resection of embolized liver. At the end of PS-TSH there are two livers: one on the right constituted by S6, and one on the left constituted by S2/S3. Abbreviations: PS: parenchyma sparing; TSH: two-stage hepatectomy; SS-PVE: super-selective portal vein embolization; GP: glissonean pedicle; FLR: future liver remnant; MHV: middle hepatic vein; MLP: mesohepatectomy for liver partition; S: segment.

The TWO-STAGE major PSH with SS-PVE is proposed when preservation of a second-order GP is planned and FLR volume is less than 40%. The first-stage procedure is MLP with resection of CRLM at the hepatocaval confluence and in one of the two livers. After this first stage, the SS-PVE is performed in the liver with CRLM still in place. At stage two, the embolized liver and all residual CRLMs are resected with a vessel-guided parenchyma-sparing technique. At the end of the second stage, both livers are preserved. The TWO-STAGE hepatectomy is defined as parenchyma sparing (PS) because it aims at preserving a single liver segment in the right liver instead of performing extended right hepatectomy. See Figure 1.

### 2.5. PS e-ALPPS (Parenchyma-Sparing Enhanced ALPPS)

Enhanced ALPPS (e-ALPPS) is proposed when first-order GP resection is planned and FLR volume is less than 40%. The first step is the MLP with resection of CRLM in one of the two livers, associating the ligature of all the portal branches of the liver with CRLM still in place, which is subsequently called the *deportalized liver*. The extrahepatic inflow and outflow vessels of the deportalized liver are tapered on elastic vessel loops left in the abdomen and fixed at the anterior wall to facilitate the second surgical step. This first step of ALPPS is defined as *enhanced* because it is not a liver partition with a single transection line as for classical ALPPS [6] but it is a mesohepatectomy with two transection lines leaving deportalized liver well perfused without any parenchymal debris and first-order GPs are completely exposed and skeletonized. Enhanced ALLPS is defined parenchyma sparing (PS) when S4 is preserved in FLR [20] or in deportalized liver and best outflow is assured using communicating veins or vascular reconstructions if necessary. In the second stage the deportalized liver is resected.

### 2.6. Staged e-ALPPS (Staged Enhanced ALPPS): Deportalized Liver Parenchyma-Sparing Resection as Intermediate Stage

**Figure 2:**
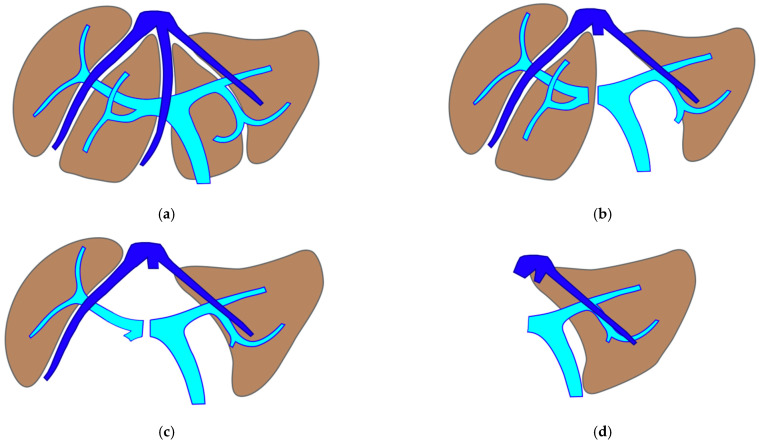
Staged e-ALPPS. The staged enhanced ALPPS is proposed when first-order GP resection is planned and FLR volume is less than 40%. (**a**) Whole liver. (**b**) First stage: vessel-guided mesohepatectomy of S1/S4 *en-bloc* with MHV for liver partition (MLP) with ligation of the first-order portal branch of the liver to be resected (hepatic artery only is preserved for the inflow), first-order portal branch of the FLR is preserved. (**c**) Intermediate stage: partial resection of the deportalized liver is performed when FLR does not reach an adequate volume after the first stage of e-ALPPS; in this stage, vessel-guided dissection and vascular reconstructions are used to assure R0 resection. (**d**) Third stage: one week after the intermediate stage, FLR volume is re-evaluated for the third stage to remove the remaining deportalized liver and to complete R0 e-ALPPS. Abbreviations: ALPPS: associating liver partition and portal vein ligation for staged hepatectomy; GP: glissonean pedicle; FLR: future liver remnant; MHV: middle hepatic vein; MLP: mesohepatectomy for liver partition.

This intervention is performed when FLR does not reach an adequate volume after the first stage of e-ALPPS. Vessel-guided dissection and vascular reconstructions are used to assure R0 resection for this intermediate stage. One week after FLR volume is re-evaluated for the third stage to remove deportalized liver and to complete R0 e-ALPPS. See Figure 2.

### 2.7. Resection of the Primary and Staged Liver Resections

When CRLMs are synchronous with the primary in place, there is no consensus about the timing of the primary resection, i.e., “primary first” versus “liver first” versus simultaneous strategy. When a staged liver resection is required, we prefer a simultaneous resection of the primary with CRLM: when the primary is in the right colon, right hemicolectomy is performed at the first hepatectomy stage, when the primary is in left colon, left hemicolectomy is performed at the last hepatectomy stage.

### 2.8. Eligibility Criteria

Among all patients admitted at our Unit from December 2008 to May 2023, we considered all consecutive patients treated with liver partition for CRLM located at the hepatocaval confluence. MLP and staged procedures were developed by adapting our previous technical advances [11] and were planned at the time of preoperative imaging. Major PSH combined with a liver augmentation procedure is identified by the need of a first/second-order GP resection to ensure R0 radicality with a FLR volume lower than 40% [11]. Eligibility for surgery required preoperative normal liver function. Liver function was assessed by routine blood tests including parameters of synthesis, cytolysis and cholestasis. This is a retrospective review of prospectively collected data conducted in accordance with the Declaration of Helsinki in which each consecutive patient was enrolled prospectively after signing a written informed consent, allowing physicians to administer the proposed treatment, perform the appropriate interventions and collect all the data in a site-specific database. None of the authors declared any potential conflict of interest.

### 2.9. Preoperative Work-Up

Preoperative imaging consisted of abdominal ultrasonography, total-body computed tomography (CT) and magnetic resonance (MR). Patients were selected for PSH after review by a multidisciplinary board including liver surgeons, medical oncologists and radiologists.

### 2.10. Hepatic Volume Calculation 

All patients underwent CT examinations to evaluate the total hepatic and the future liver remnant (FLR) volume before extensive liver resection. FLR volume < 40% was considered insufficient in patients treated with chemotherapy. CT scans were performed to evaluate FLR volume. CT scan protocol (Discovery CT750 HD system; GE Healthcare, Wauwatosa, WI, USA) included an unenhanced scan followed by triphasic acquisition after injection of non-ionic contrast medium followed by 40 mL saline flush using an automatic injector (MEDRAD Stellant; MEDRAD, Inc., Warrendale, PA, USA).

Post-contrast imaging scan was determined by automated bolus triggering (SmartPrep; GE Healthcare).

On an independent workstation (Advantage Windows 4.7, GE Healthcare) the interventional radiologist and the liver surgeon calculated the total hepatic and the FLR volume using a dedicated semi-automated CT software. 

Only axial slices of at least 1.25 mm in portal venous phases were considered reliable to calculate hepatic volumes. Main portal extrahepatic vein, gallbladder and any metastasectomy area were manually excluded.

Once the segmentation was completed the software automatically calculated the hepatic and FLR volume (mL).

### 2.11. Operative Technique 

A J-shaped laparotomy, eventually extended to a U-shaped thoracoabdominal laparotomy [21], was performed. Xipho-pubic incision was preferred in the case of simultaneous resection of the left colon. A complete abdominal exploration was performed to identify peritoneal or nodal lesions in the hepatoduodenal ligament or intestinal mesentery. If additional nodal or peritoneal lesions were found, liver resection was performed only if all the newly discovered lesions were deemed resectable. IOUS was performed using a BK Medical-mod 2202-7 ProFocus Ultraview (Herlev, Denmark). The contact between HV/GP and CRLM was confirmed on IOUS findings. The decision to resect HV or GP was based on vessel contact only, independently from the amount of circumferential relationship or evidence of endothelial discontinuation or biliary dilation. 

The liver was mobilized by dividing the right and left triangular and coronary ligaments to properly control the hepatocaval confluence. Hepatic veins were always tapered and clamped when necessary. Parenchymal transection was carried out under intermittent pedicle clamping by means of crush clamping, ligatures and bipolar electrocautery for thinner vessel coagulation. With the increasing complexity of the cases, especially for vascular reconstructions, longer durations of clamping became inevitable; thus, a reperfusion time equal to the clamping time was adopted in accordance with the anesthesiology team. The HVs and/or GP were resected *en-bloc* with CRLM. MLP was always extended to the paracaval portion of S1 and/or caudate processus of S1. MHV is usually resected to assure the complete liver partition. If the right hepatic vein (RHV) and/or left hepatic vein (LHV) should be resected *en-bloc* with CRLM, the HVs were skeletonized to obtain an adequate length for a direct reconstruction with an end-to-end anastomosis with the other end of the same HV. If necessary, HV could be reconstructed by interposition of a ringed polytetrafluoroethylene (PTFE) 7 mm graft [11]. Extracorporeal circulation was never considered. At the end of the intervention, the specimen volume was calculated by immersion in saline solution. A video describing the vessel-guided liver partition procedure is available elsewhere [22].

The pathologist marked with ink the cut surface of the liver specimen, and the absence of hepatocyte layer/vessel wall between tumor and the ink was considered a 0 mm margin (R1 resection). Histopathological growth patterns [23] and histological tumor regression grade (TRG) assessment [24] in patients treated with neo-adjuvant chemotherapy were evaluated.

### 2.12. Patient Follow-Up 

After PSH, a tight follow-up was scheduled every 2 months with physical examination, complete blood profile, CEA, CA 19-9 and CT of the chest and abdomen during the first year, every 4 months for the second and the third year and every 6 months thereafter. The cut-off date for analyses was 30 June 2023.

### 2.13. Statistics 

Relapse-free survival (RFS) was calculated from the day of surgical resection to the evidence of disease relapse or death from any cause. Overall survival (OS) was calculated from the day of surgical resection until death from any cause. Survival curves were estimated by the Kaplan–Meier method and carried out with MedCalc Statistical Software 19.4.1 (https://www.medcalc.org, accessed on 30 June 2023).

## 3. Results

**Figure 3:**
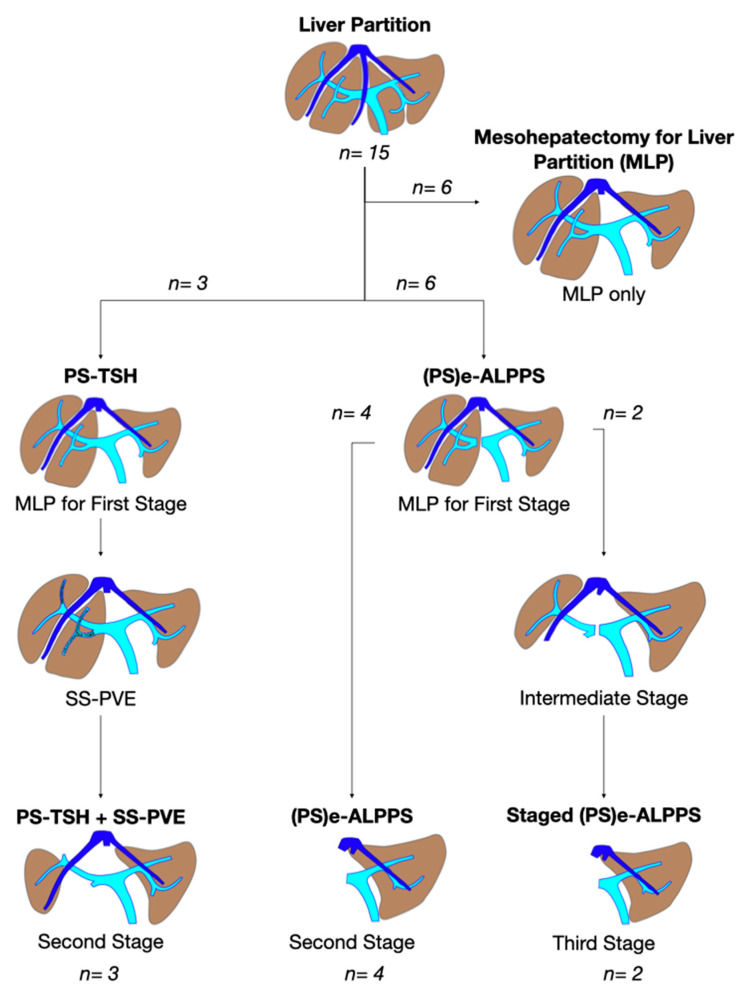
Diagram showing the treatment of 15 consecutive cases of mesohepatectomy for liver partition (MLP). Six cases were treated with MLP only and nine cases required liver augmentation techniques due to insufficient FLR volume. Liver augmentation was obtained with super-selective PVE in three cases and with enhanced ALPPS in six cases. Two cases of enhanced ALPPS required a further intermediate step with resection of the deportalized liver in order to achieve an adequate FLR. TWO-STAGE hepatectomy was defined as parenchyma sparing because it aims to preserve a single segment in the right liver instead of performing extended right hepatectomy. ALPPS is defined as enhanced because liver partition is not performed with a single transection line, as described for classical ALPPS, but it consists in a mesohepatectomy with two full thickness transection lines. ALPPS is defined parenchyma sparing when S4 is preserved. Abbreviations: MLP: mesohepatectomy for liver partition; FLR: future liver remnant; PVE: portal vein embolization; TS-PSH: two-stage parenchyma-sparing hepatectomy; ALPPS: associating liver partition and portal vein ligation for staged hepatectomy; SS-PVE: super-selective PVE; e-ALPPS: enhanced ALPPS; PS: parenchyma sparing.

From December 2008 to May 2023 a total of 443 liver resections for CRLM were performed at the General Surgery Unit of the University Hospital of Pisa, 180 (40.6%) PSHs for CRLM deep located in segments S1-S4a-S7-S8. According to the inclusion criteria specified above, between December 2012 and May 2023, 15 (3.3%) consecutive vessel-guided MLP were performed (M/F ratio 1:2). The diagram in Figure 3 depicts the specific procedures performed in the 15 consecutive cases.

Patients’ median age was 58 years (range 39–77). All patients had a preoperative normal liver function. All patients but one had synchronous CRLM. Ten patients were node-positive N of the time of primary tumor resection, which was located in the left colon in 11 cases, in the right colon in 3 and in the rectum in 1. Four tumors were RAS mutated and 11 were RAS and BRAF wild type.

The median number of CRLMs was 11 (range: 0–67, one patient had a complete radiological response). Median diameter of the largest CRLM was 35 mm (range 0–80 mm). CRLMs were bilobar in 11 (73%) patients. All patients had received preoperative systemic therapy, including a doublet or triplet of cytotoxics combined in all cases except one with a biologic agent, either bevacizumab or an anti-epidermal growth factor receptor monoclonal antibody. An immune checkpoint inhibitor (avelumab) was added in three cases in the frame of a prospective clinical trial. The median number of preoperative chemotherapy cycles was eight (range: 3–47). Two patients experienced stable disease, ten patients partial response and one complete response during the preoperative systemic therapy. Two patients underwent surgery following a modest dimensional increase of CRLMs that were judged unresectable in other institutions following clear and durable RECIST responses to the administered chemotherapy. One patient had already undergone a previous e-OSH for the resection of 20 CRLMs and experienced a local right first-order GP recurrence with biliary infiltration at the R1-vascular liver cut surface.

### 3.1. MLP

**Figure 4:**
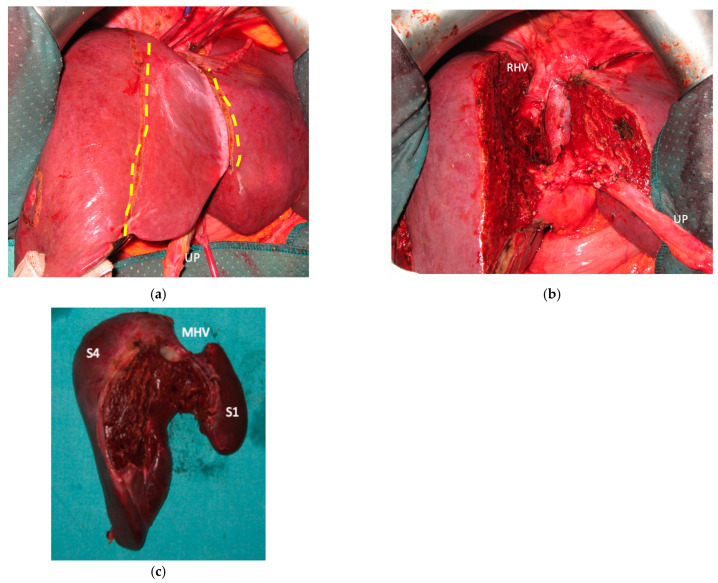
Intraoperative field images of a vessel-guided mesohepatectomy of S1/S4 *en-bloc* with MHV for liver partition (MLP). MLP is a minor resection (less than 3 adjacent liver segments) inducing a major hepatic scaffold modification transforming the liver into a paired organ. (**a**) Liver transection lines (yellow dotted lines). (**b**) Intraoperative view of the two livers. (**c**) Surgical specimen of S4/S1 *en-bloc* with MHV corresponding to a volume of 205 mL. Abbreviations: MLP: mesohepatectomy for liver partition; MHV: middle hepatic vein; RHV: right hepatic vein; S: segment; GP: glissonean pedicle; UP: umbilical portion.

In all 15 cases planned vessel-guided MLP was successfully carried out. For the surgical approach, a J-shaped laparotomy was used in 13 cases and the xipho-pubic incision in two; no thoraco-abdominal incision was needed. In two cases of MLP performed as the first stage of ALPPS, liver partition was obtained with a single transection line: in one case (FLR = S2/S3/S4b) between a part of S4b and S5 since S4a/S1 were resected in previous e-OSH, in the other case (FLR = S4/S2/Spiegel lobe) the single transection line preserved part of S4a/S4b and the Spiegel lobe. In 11 cases liver partition was obtained with a minor mesohepatectomy: in one case with resection of S4a/S1 partially extended to S5 preserving S4b, in two cases with resection of S1/S4 preserving the Spiegel lobe, in one case with resection of segments S5/S8 extended to the caudate processus of S1 preserving the Spiegel lobe and paracaval portion of S1 and in the other seven cases with anatomic resection of S1/S4 (see Figure 4).

**Figure 5:**
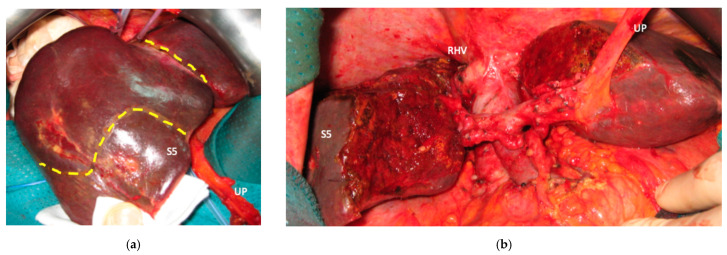
This is a case of liver partition obtained with a major mesohepatectomy performed for CRLM that behaves as biliary cancer infiltrating the confluence of left and right biliary ducts up to S8 duct; this is a highly challenging location for CRLMs at high risk of worsening patient’s quality of life and hampering chemotherapy administration due to jaundice; for this reason, liver resection was performed despite a modest dimensional increase of CRLMs after several courses of FOLFIRI + Bevacizumab administered over three years and after an initial complete radiological response. (**a**) Intraoperative field with the identification of the two liver transection lines for liver partition (yellow dotted lines). (**b**) Liver partition at the end of resection of S1/S4/S8 *en-bloc* with extrahepatic biliary tree resected up to left duct, S5 duct and posterior duct. Three-duct jeujunostomy was performed. Thirty-eight months after liver partition, the patient is under chemotherapy treatment (no jaundice) with stable and asymptomatic extrahepatic and hepatic recurrence. Abbreviations: CRLM: colorectal liver metastases; S: segment; RHV: right hepatic vein; UP: umbilical portion.

Liver partition was obtained with a major mesohepatectomy in two cases with anatomical resection of S1/S4/S8 (*en-bloc* with biliary tree in one, see Figure 5).

In nine patients additional liver resections were performed (median 1; range 1–5); in seven patients additional resections involved S2 and S3, in four patient mesohepatectomy was partially extended to S2/S3. Middle hepatic vein was resected in twelve cases, preserved in two and reconstructed in one with an end-to-lateral anastomosis with LHV. Median liver volume resected was 223 mL (range 0–480). Surgery lasted a median of 650 min (range 385–965), median intermittent clamping time was 132 min (range 22–304), median liver cut surface was 225 cm^2^ (range 50–409), median blood loss was 350 cc (range 100–3300), 12 (80%) patients were transfused with a median of 3 blood units (range 2–4). Seven (47%) patients had an uneventful postoperative course, two patients had a grade I complication, three a grade II complication, one a grade IIIa complication (pleural effusion treated with thoracentesis) and two (13%) a grade IIIb (hemoperitoneum), no grade IV or V complications occurred. Liver function tests returned to normal levels within the fifth post-operative day in all patients. One patient developed an asymptomatic portal vein thrombosis localized at the common trunk. One patient developed ascites (first step of e-ALPPS with FLR = part of S6/S7), no biliary leaks occurred.

The median duration of the Intensive Care Unit stay was 2 days (range 1–62) with a median overall hospital stay of 14 days (range 6–62). Three patients completed the ALPPS procedure within a single hospital admission.

### 3.2. Staged Major Parenchyma-Sparing Hepatectomy and Liver Augmentation Techniques

In 9 (60%) patients with a median of 23 (range 9–67) CRLMs, MLP was the first stage of a programmed major parenchyma-sparing staged procedure associated to a liver augmentation procedure. Planned treatment was completed in all the cases. Median FLR volume increased from a median of 15% (range: 7–20%) up to 41% (range: 37–69%).

### 3.3. PS-TSH with SS-PVE

**Figure 6:**
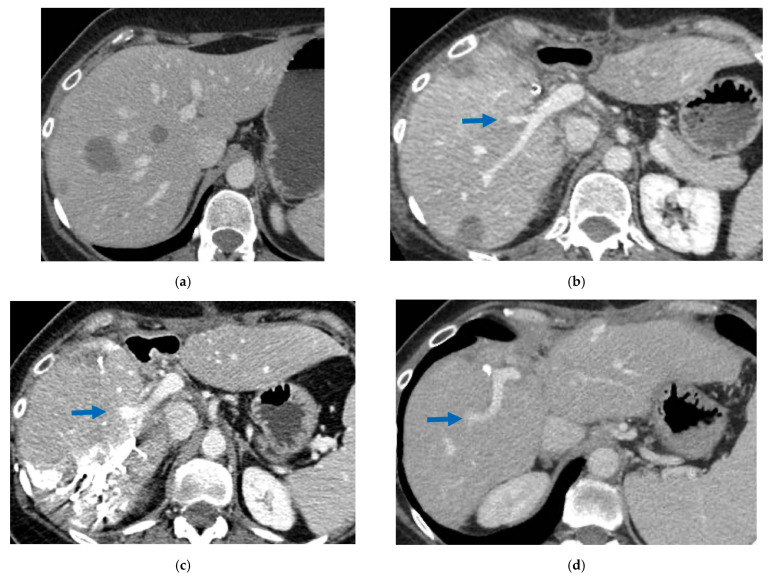
CT Images of PS-TSH with SS-PVE performed to treat 35 bilateral CRLMs. (**a**) Partial response after FOLFOXIRI + Bevacizumab 12 cycles; FLR (=S2/S3) volume is 15%. (**b**) Blue arrow points GP 5 after the first stage consisting of resection of S1/S4 extended to S2/S8/S5 plus metastasectomy of S2 with tangential resection of LHV. (**c**) Fourteen days after stage 1 SS-PVE was performed on the right liver preserving GP 5 (blue arrow); 37 days after SS-PVE FLR (=S2/S3/S5) increased up to 37%. (**d**) Blue arrow points GP 5. Twenty-five months after PS-TSH with SS-PVE the patient is disease free; the liver’s only disease recurrence was successfully treated with another 3 liver resections (2 on the right liver and 1 on the left). Abbreviations: PS-TSH: parenchyma-sparing TWO-STAGE hepatectomy; SS-PVE: super-selective portal vein embolization; CRLM: colorectal liver metastases; FLR: future liver remnant; S: segment; GP5: glissonean pedicle for segment 5; LHV: left hepatic vein.

**Figure 7:**
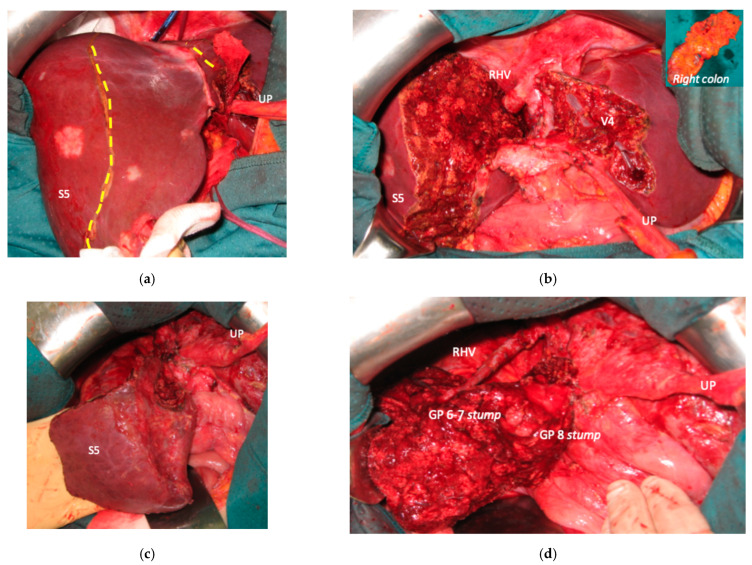
Intraoperative field images of PS-TSH with SS-PVE performed to treat 35 bilateral CRLMs. (**a**) Hole liver at the first stage after FOLFOXIRI + Bevacizumab 12 cycles, with liver transection lines (yellow dotted lines). (**b**) At the end of the first stage of TS-PSH with SS-PVE: resection of S1/S4 extended to S2/S8/S5 plus metastasectomy of S2 with tangential resection of LHV, first-order GPs are completely exposed; the primary is resected with a simultaneous right hemicolectomy. (**c**) At the end of the second stage performed 58 days after the first stage, embolized liver is completely resected and S5 only is preserved, the left liver remains untouched. (**d**) At the second stage S5 was twisted to avoid kinking of RHV which is completely skeletonized, for this reason GPs 6–7 and GP 8 appear inverted. Abbreviations: PS-TSH: parenchyma-sparing TWO-STAGE hepatectomy; SS-PVE: super-selective portal vein embolization; CRLM: colorectal liver metastases; RHV: right hepatic vein; LHV: left hepatic vein; V4: scissural hepatic vein; S: segment; GP: glissonean pedicle; UP: umbilical portion.

**Figure 8:**
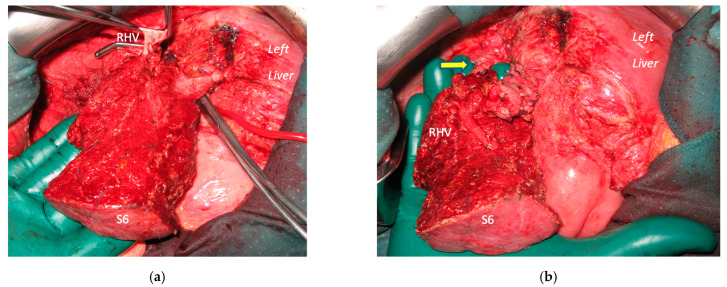
This is the intraoperative field of the second stage of a case of a vessel-guided TWO-STAGE major parenchyma-sparing hepatectomy with super-selective PVE. (**a**) Anatomic view of S6 with exposure of RHV which is the anatomic boundary of S6; RHV is sectioned at the entrance in S7/S8; anterior GP is sectioned at the origin and GP6 passes under RHV. (**b**) RHV is reconstructed with an end-to-end anastomosis (yellow arrow). This two-stage hepatectomy is parenchyma sparing since S6 is preserved (the second stage of classical TSH is usually a right extended hepatectomy); this second stage, despite the technical complexity, is well tolerated by the patient since the left liver remains untouched during the second stage. Abbreviations: PVE: portal vein embolization; S: segment; RHV: right hepatic vein; GP: glissonean pedicle; TSH: two-stage hepatectomy.

Super-selective portal vein embolization was performed in three patients since the resection of first-order GPs was deemed not necessary and a TWO-STAGE major PSH with SS-PVE was planned. Percutaneous PVE was achieved under US guidance via an ipsilateral approach through the tumor-bearing liver in two patients and via a contralateral approach through the future remnant liver in one patient. The accesses were respectively by S6, S5 and S3 portal vein branches under US guidance and local anesthesia. In all three cases S1/S4 were anatomically resected *en-bloc* with MHV at the first stage (MLP). Super-selective PVE consisted in embolization of portal branch for S6/S7/S8 in one case, for S7/S8 in one case and for S5/S8 in one case. The second stage was performed 58, 57 and 63 days after the first stage and consisted in the resection of S6/S7/S8 with RHV skeletonization in one case (see Figure 6 and Figure 7), in resection of S7/S8 partially extended to S5/S6 *en-bloc* with RHV reconstructed with an end-to-end anastomosis in one case (see Figure 8) and in resection of S7/S8/S5 *en-bloc* with RHV reconstructed with end-to-end anastomosis in another case. No complications occurred after surgery and patients were discharged from the hospital on the 11th, 11th and 17th post-operative day. The primary was resected at the time of the first stage (right hemicolectomy) in two cases and before the first stage (left hemicolectomy, “primary first” strategy) in one case.

### 3.4. Staged (PS) e-ALPPS

**Figure 9:**
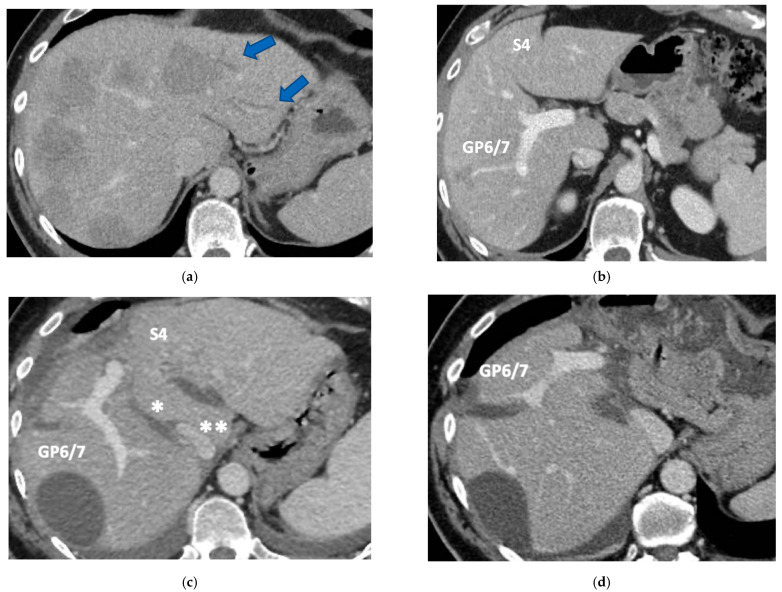
CT Images of PS e-ALPPS performed to treat 19 bilateral CRLMs infiltrating the left first-order glissonean pedicle; FLR is represented by a part of S6 and S7. (**a**) CT at the diagnosis: arrows indicate biliary dilation due to infiltration of left first-order glissonean pedicle. (**b**) Partial response after FOLFIRI + Cetuximab 7 cycles; FLR (=part of S6/S7) volume is 17%; segment 4 (S4) and posterior glissonean pedicle (GP 6/7) are indicated. (**c**) Forty-one days after the first stage FLR (=part of S6/S7) volume increases up to 41%; the first stage of e-ALPPS consisted in resection of S5/S8/caudate processus partially extended to S6/S7 with metastasectomy of S7; this case of e-ALPPS is defined as parenchyma sparing since MHV and S4 were preserved in the deportalized liver (S1-S2-S3-S4); * paracaval portion of S1; ** Spiegel lobe. (**d**) Four and a half months after the PS e-ALPPS the patient is disease free. Abbreviations: PS: parenchyma sparing; e-ALPPS: enhanced ALPPS; CRLM: colorectal liver metastases; FLR: future liver remnant; S: segment GP 6/7: posterior glissonean pedicle for S6/S7; MHV: middle hepatic vein; * paracaval portion of S1; ** Spiegel lobe.

In six patients the resection of first-order GP was planned and e-ALPPS was performed. Four e-ALPPS were parenchyma sparing since a part of S4 was preserved (in deportalized liver in one case of FLR = part of S6/S7, see Figure 9).

**Figure 10:**
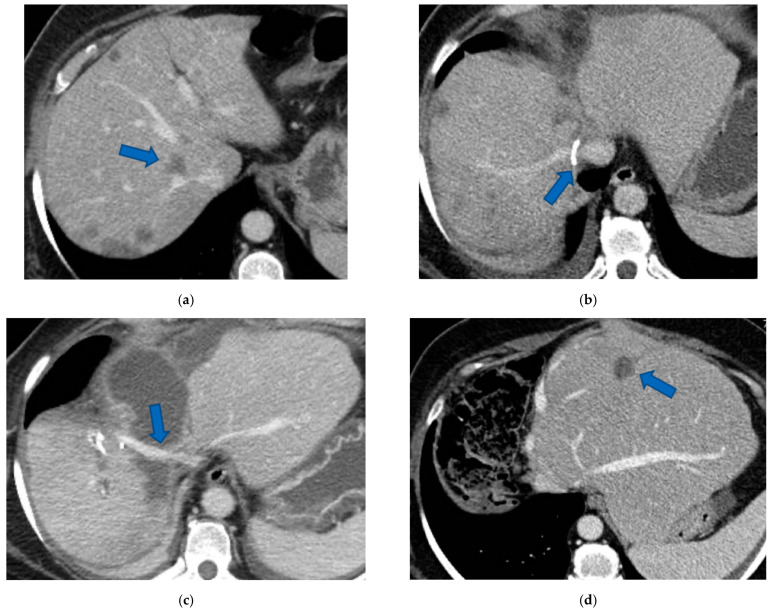
CT Images of staged e-ALPPS performed to treat 55 bilateral CRLMs infiltrating RHV and MHV. (**a**) Partial response after FOLFOXIRI + Panitumumab 8 cycles; FLR (=part of S2/S3) volume is 7%; blue arrow indicates CRLM infiltrating RHV and MHV at the hepatocaval confluence. (**b**) Seventeen days after the first stage (resection of S1/S4 *en-bloc* with MHV and extended to S2/S3; RHV is suspended on a vessel loop; FLR (=part of S2/S3) volume increases up to 28% and an intermediate surgical stage for partial resection of deportalized liver is planned to boost liver regeneration; blue arrow indicates elastic vessel loop around RHV. (**c**) Seven days after intermediate stage (resection of S5/S8 *en-bloc* with RHV reconstructed with end-to-end anastomosis) FLR (=part of S2/S3) volume increases up to 41% and the third stage is planned to complete the staged e-ALPPS; blue arrow indicates reconstructed RHV. (**d**) Nineteen months after staged e-ALPPS the patient is disease free; a CRLM was treated by percutaneous ablation (blue arrow) 5 months after staged e-ALPPS. Abbreviations: e-ALPPS: enhanced ALPPS; CRLM: colorectal liver metastases; RHV: right hepatic vein; MHV: middle hepatic vein; FLR: future liver remnant; S: segment.

**Figure 11:**
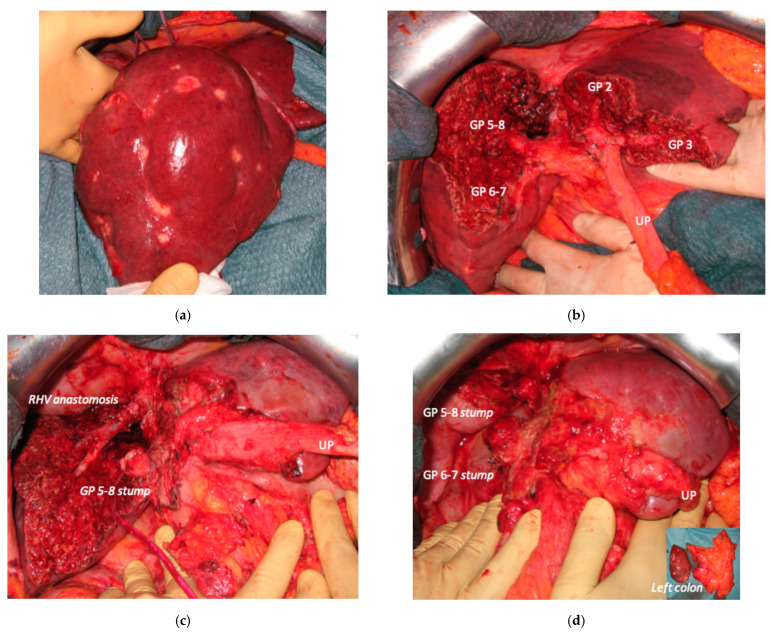
Intraoperative field images of staged e-ALPPS performed to treat 55 bilateral CRLMs infiltrating RHV and MHV. (**a**) Hole liver at the first stage after FOLFOXIRI + Panitumumab 8 cycles. (**b**) At the end of the first stage of e-ALPPS: resection of S1/S4 partially extended to S2/S3 *en-bloc* with MHV plus 3 metastasectomies in S2/S3 and section between ligatures of right portal vein, first-order GPs are completely exposed. (**c**) At the intermediate stage of e-ALPPS 19 days after the first stage for partial resection of deportalized liver: R0 resection of S5/S8 *en-bloc* with RHV reconstructed with end-to-end anastomosis. (**d**) At the third stage of e-ALPPS eight days after the intermediate stage: resection of S6/S7 and simultaneous resection of the primary with a left hemicolectomy. Abbreviations: e-ALPPS: enhanced ALPPS; CRLM: colorectal liver metastases; RHV: right hepatic vein; MHV: middle hepatic vein; S: segment; GP: glissonean pedicle; UP: umbilical portion.

**Figure 12:**
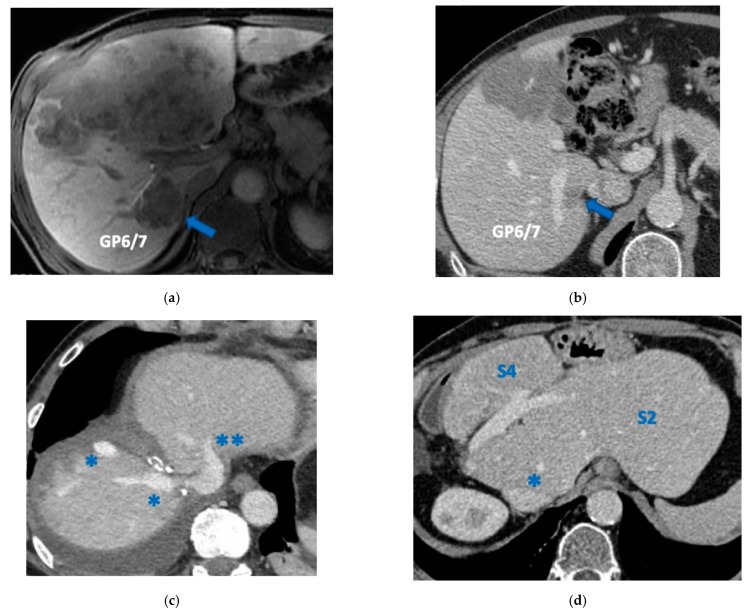
MR and CT images of staged PS-ALPPS performed to treat 16 bilateral CRLM infiltrating the anterior and posterior glissonean pedicle. (**a**) Hepatobiliary phase of magnetic resonance at diagnosis, blue arrow indicates the CRLM in contact with the posterior glissonean pedicle (GP6/7), posterior bile duct is regular but with a branch inside the CRLM. (**b**) Partial response after FOLFOXIRI + Cetuximab + Avelumab 8 cycles; blue arrow indicates the CRLM in contact with GP6/7 with massive shrinkage after chemotherapy; we decided to not propose the e-OSH with detachment from GP6/7 preferring the R0 resection; a PS-ALPPS was planned since FLR (=S2/part of S4/Spiegel lobe) volume was 15%; two weeks after the first stage (transaction line preserving part of S4a/S4b and Spiegel lobe and resection of S3, section of MHV and section of right portal vein) FLR increased up to 29% but was judged not enough and an intermediate stage was planned. (**c**) CT 7 days after intermediate stage (resection of S5/S8), FLR volume increased up to 39% and the third stage was planned to complete the R0 staged PS-ALPPS with simultaneous resection of the primary (left hemicolectomy); * indicates RHV; ** indicates LHV. (**d**) CT after staged PS-ALPPS showing S4, S2 and Spiegel lobe indicated by the single asterisk; 15.5 months after staged PS-ALPPS the patient is disease free; the ALPPS is defined parenchyma sparing since S4 is preserved; * Spiegel lobe. Abbreviations: PS-ALPPS: parenchyma-sparing ALPPS; CRLM: colorectal liver metastases; GP6/7: posterior glissonean pedicle for segments 6 and 7; FLR: future liver remnant; RHV: right hepatic vein; LHV: left hepatic vein; MHV: middle hepatic vein; S: segment.

**Figure 13:**
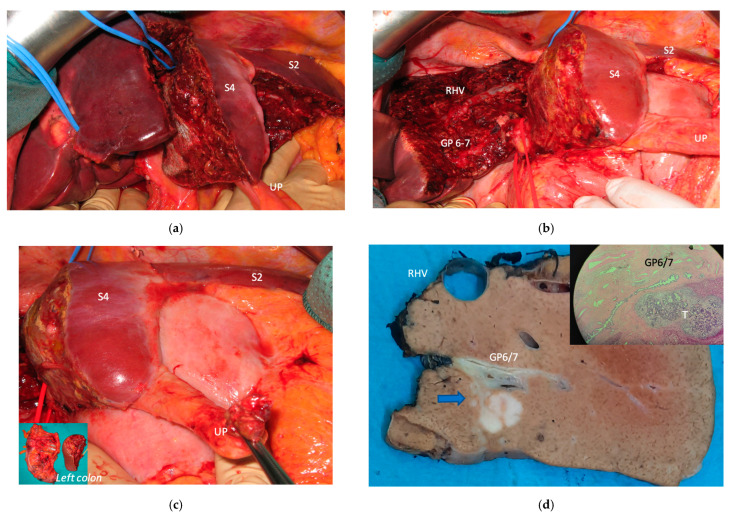
Intraoperative field images of staged PS-ALPPS performed to treat 16 bilateral CRLMs infiltrating the anterior and posterior glissonean pedicle. (**a**) At the end of the first stage, in this case liver partition was obtained with a single transection line preserving a part of S4 and Spiegel lobe; FLR = S2/S4/Spiegel lobe, FLR volume was 17%. (**b**) At the end of the intermediate stage consisting in the anatomical resection of S5/S8; this intermediate stage of partial resection of the deportalized liver was necessary because FLR increased up to 26% two weeks after the first stage was judged not enough to complete the PS-ALPPS. (**c**) At the third stage of this R0 staged PS-ALPPS; at this stage was performed the simultaneous resection of the primary (left hemicolectomy). (**d**) Gross appearance of CRLM in contact with the posterior glissonean pedicle (GP6/7), blue arrow indicates satellite nodules of tumor around GP6/7; in the picture in picture the hematoxylin and eosin 10× magnification of a satellite nodule infiltrating GP6/7. Abbreviations: PS-ALPPS: parenchyma-sparing ALPPS (the ALPPS is defined PS because S4 is preserved); CRLM: colorectal liver metastases; GP6/7: posterior glissonean pedicle for segments 6 and 7; FLR: future liver remnant; RHV: right hepatic vein; S: segment.

In four cases the Spiegel lobe was preserved. At the first stage (MLP) MHV was resected in four cases, preserved in deportalized liver in the case of FLR = part of S6/S7 and reconstructed with an end-to-lateral anastomosis with LHV (FLR = S4b/S2/Spiegel lobe) in one case. In two patients (FLR 15% = part of S4/S2 and Spiegel lobe; FLR 7% = part of S2/S3) FLR volume was deemed inadequate after the first step of e-ALPPS (FLR 29% and 28%, 15 and 17 days after the first stage), daily liver growth after the first stage was 21 mL/day in the first 8 days and 8 mL/day in the subsequent 7 days in one case and 19 mL/day in the first 17 days in the other (in the latter, growth kinetic cannot be evaluated during the first week since CT was not performed). Intermediate stage of partial resection of the deportalized liver was performed (in both cases resection of S5-S8, in one case *en-bloc* with RHV which was reconstructed with an end-to-end anastomosis). Seven days after the intermediate stage FLR volume increased up to 39% and 41% with a daily growth of 18 mL/day and 25 mL/day. The third stage to complete the ALPPS procedure was performed (23 and 27 days after the first stage) resecting the last part of deportalized liver (S6/S7). In both cases the left hemicolectomy was associated to third step of staged e-ALPPS (see Figure 10, Figure 11, Figure 12 and Figure 13).

In the other four cases the primary was resected at the time of the first stage in one case (right hemicolectomy), after e-ALPPS procedure (“liver first” strategy) in one case and before e-ALPPS procedure (“primary first” strategy) in two cases.

In the other four cases of e-ALPPS the second stage was performed 7, 8, 30 and 54 days after the first stage. All patients were discharged a median of 18 (range 4–66) days after the last surgery. One patient had severe liver failure and died at 2.3 months (this patient developed an asymptomatic thrombosis of the common trunk of the portal vein after e-ALPPS stage 1 with FLR increase up to 39% after therapy with heparin). One patient developed ascites associated to transient deficit of liver synthesis function due to insufficient oral caloric intake recovered after enteral nutrition (staged e-ALPPS with FLR = part of S2/S3). One patient developed a delayed subphrenic abscess at 1.5 months.

### 3.5. Surgical Reconstructions

Biliary tract was reconstructed in two cases. In one case of MLP of S1/S4/S8 extrahepatic biliary tract was resected up to b5, left duct and posterior duct (Figure 5) and reconstructed with a three-duct jeujunostomy. In the other case, left-duct jeujunostomy was performed at the first stage of e-ALPPS simultaneously with portal vein resection and reconstruction with an end-to-end anastomosis.

In a case of e-ALPPS an intermediate stage of PSH of the deportalized liver was necessary (due to inadequate FLR volume of S2/S3 18 days after the first stage), at the intermediate stage S5/S8 were resected *en-bloc* with RHV which was reconstructed with an end-to-end anastomosis (Figure 11). 

In a case of e-ALPPS at the first stage, S4b was preserved and MHV was reconstructed with an end-to-lateral anastomosis with LHV.

In two cases of TWO-STAGE vessel-guided major PSH with super-selective PVE at the second stage RHV was reconstructed with an end-to-end anastomosis since RHV was resected with S7/S8 partially extended to S5/S6 in one case and with S7/S8/S5 in another case (Figure 8).

### 3.6. Histology

One MLP (resection of S1/S4a) was R1, all other liver resections including the intermediate stage of e-ALPPS and all the second/third stages were R0. 

In seven cases the histopathological growth pattern was assessed as replacement/pushing pattern, replacement in one case, pushing in one case, desmoplastic in three cases and non-definable in three cases.

In the 15 chemotherapy pre-treated patients TRG was reported: TRG4 in six cases, TRG3 in four cases, TRG2 in three cases and TRG1 in two cases. For the patient with complete radiological response at histology the CRLM disappeared but, by performing an anatomical resection of S1/S4 *en-bloc* with MHV, the vanishing CRLM was definitely resected even if no disease was found in the surgical specimen. 

First-order GPs were infiltrated in four cases and HVs at the hepatocaval confluence in three cases.

### 3.7. Follow-Up

**Figure 14:**
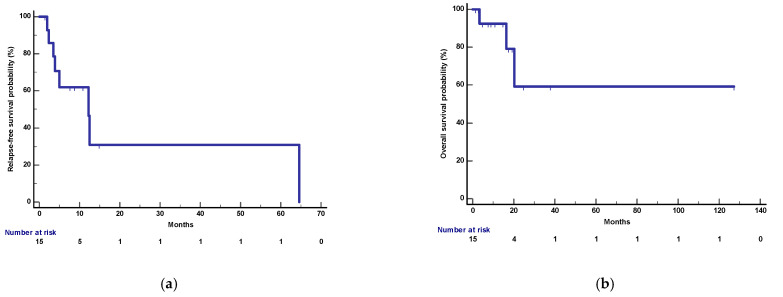
Relapse-free survival (RFS, blue line) was calculated from the day of surgical resection to the evidence of disease relapse or death from any cause. Overall survival (OS, blue line) was calculated from the day of surgical resection until death from any cause. Survival curves were estimated by the Kaplan–Meier method and carried out with MedCalc Statistical Software 19.4.1 (https://www.medcalc.org, accessed on 30 June 2023). After a median follow-up of 17.5 months (95%CI: 4.8–127 months), 3 deaths and 8 relapses were recorded. (**a**) RFS: Median RFS was 12.2 months (95%CI: 3.4–64.6) and 1-year RFS was 62%. (**b**) OS: Median OS was not reached and 1-year OS was 92%.

After a median follow-up of 17.5 months (95%CI: 4.8–127 months), 3 deaths and 8 relapses were recorded. Median OS was not reached and 1-year OS was 92%. Median RFS and 1-year RFS were 12.2 months (95%CI: 3.4–64.6) and 62%, respectively (Figure 14).

Seven patients are currently disease free: three of them following MLP (11.5, 5.5 and 2 months after surgery), two after PS-TSH with SS-PVE (at 9.5 and 8.2 months), one after staged e-ALPPS (at 15.5 months) and one after e-ALPPS (at 4.2 months). 

Another three patients are disease free after the locoregional treatment of disease recurrence: one patient underwent two subsequent re-resections and is currently disease free more than 10 years after MLP [25], one patient underwent three subsequent re-resections and is disease free more than two years after PS-TSH with SS-PVE (performed for 35 synchronous CRLMs simultaneously with right hemicolectomy), one patient was treated with percutaneous ablation and is disease free 19 months after staged e-ALPPS (performed for 55 synchronous CRLMs simultaneously with left hemicolectomy).

Two out of eight patients with unresectable disease relapse are still alive although with evident disease: one patient 18 months after e-ALPPS performed as a “liver first” strategy for 27 synchronous CRLMs with left colon adenocarcinoma and interaortocaval lymph nodes, then followed by a left hemicolectomy and interaortocaval lymphadenectomy, who experienced an extrahepatic recurrence 3 months after surgery; and one patient 38 months after a major MLP associated with a three-duct jeujunostomy (performed for two CRLMs infiltrating the confluence of left and right bile ducts up to S8 bile duct and with modest dimensional increase of CRLM after 47 cycles of FOLFIRI + Bevacizumab across several reintroductions, Figure 5), with hepatic and extrahepatic recurrence 12 months after surgery. 

**Figure 15:**
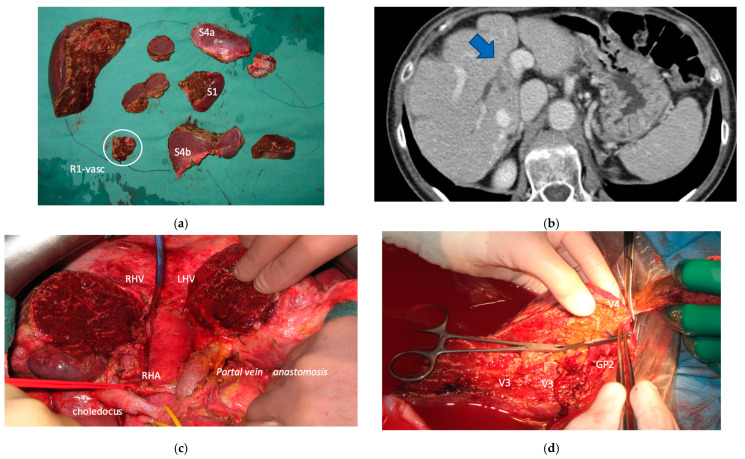
This is a very complex case treated by our MDT. (**a**) Twenty CRLM were resected with an e-OSH, 1 CRLM was detached from right first-order GP (R1-vasc), S1 and a part of S4a/S4b were resected. (**b**) Four months after e-OSH disease recurred at the first-order right GP (at the site of previous R1-vascular resection, blue arrow) with infiltration of the biliary duct; this is the worst place since it behaves as a biliary neoplasm. (**c**) Intraoperative field of the first stage of PS-ALPPS (a part of S4 was preserved) performed for R0 resection after disease stability obtained with 8 cycles of FOLFOXIRI + Bevacizumab and other 13 cycles of maintenance chemotherapy; portal vein was resected and reconstructed with an end-to-end anastomosis; left bile duct was reconstructed with an hepaticojeujunostomy. (**d**) Intraoperative field of monosegmental liver autotransplantation performed to treat a disease recurrence occurred 8 months after ALPPS and located at S2 infiltrating GP2 and LHV; the resection of S2 *en-bloc* with LHV root and GP2 was completed ex situ, a complex outflow reconstruction was performed for the residual 3 hepatic veins for S3 and S4 (V3, V3, V4). At the follow-up the patient experienced a delayed biliary leak and died of sepsis 92 days after liver autotransplantation, 16.5 months after PS-ALPPS. Abbreviations: MDT: multidisciplinary team; CRLM: colorectal liver metastases; e-OSH: enhanced one-stage hepatectomy; GP: glissonean pedicle; PS-ALPPS: parenchyma-sparing ALPPS (preservation of a part of S4); S: segment; GP2: glissonean pedicle for segment 2; LHV: left hepatic vein; V3: hepatic vein for S3; V4: hepatic vein for S4; RHV: right hepatic vein; RHA: right hepatic artery.

Three patients died. One (11%) of the nine patients who completed the liver augmentation strategy died within 90 days of completing the strategy (at 2.3 months). This patient had a diagnosis of right-sided colon cancer with 67 synchronous CRLMs and achieved stable disease after six cycles of FOLFOXIRI + bevacizumab. He was treated with an e-ALPPS, and after the first stage of MLP and simultaneous right hemicolectomy developed an asymptomatic thrombosis of the common trunk of the portal vein. After a high dose of heparin, FLR volume increased up to 39%, the second stage of e-ALLPS was complicated by severe liver failure, the patient was discharged from the hospital but died with liver failure and lung disease progression. The other two patients died due to disease recurrence. One patient with five synchronous CRLM from rectal adenocarcinoma experienced disease recurrence 2 months after surgery and died 20.5 months after MLP. The third patient had a previous e-OSH with detachment from the right GP of one of the 20 synchronous resected CRLMs. Four months after e-OSH a local disease recurrence occurred at the site of detachment with infiltration of the right biliary duct. Since disease recurrence was stable after 8 cycles of FOLFOXIRI + Bevacizumab and other 13 cycles of maintenance chemotherapy, an e-ALPPS (FLR = residual S2/S3/S4b) with portal vein and biliary resection/reconstruction was offered. Eight months after e-ALPPS, a new recurrence occurred in S2 infiltrating the LHV at the hepatocaval confluence and GP for S2. This new disease recurrence was stable after three cycles of FOLFOXIRI + Bevacizumab followed by 4 cycles of FOLFIRI + Bevacizumab. A monosegmental auto-transplantation was proposed (FLR = S3/S4b increased after e-ALPPS up to 769 mL corresponding to a graft-to-recipient body weight ratio of 1.37) and performed with success but it was complicated by a late biliary leak and the patient died of sepsis 92 days after liver auto-transplantation, 16.5 months after e-ALPPS (see Figure 15).

## 4. Discussion

Since the beginning of our experience we have been focused on the development of parenchyma-sparing liver surgery replicating the new interventions described by Torzilli et al. [1]. We have also used vascular reconstruction to spare liver parenchyma and to expand FLR volume showing advanced solutions and good results in term of short-term outcome [26] and avoiding major hepatectomies and the need for PVE and/or ALPPS and implementing e-OSH [11]. The availability of more and more efficacious systemic treatments, mainly upfront but also in subsequent lines, raised the bar of (un)resectability, allowing us to achieve relevant and/or durable tumor responses and potentially deeply affecting disease biology [27]. As a consequence, new challenges are now open for multidisciplinary teams (MDT) dealing with increasingly complex cases of CRLM. In 2020, pushing the limit of e-OSH, for the first time at our center a perioperative death occurred. Our MDT held a morbidity/mortality briefing asking how far we should push the complexity of e-OSH. We assumed we reached our limit with this technique. Since it is not recommended to perform an e-OSH simultaneously with the resection of the primary, we decided, in the presence of the primary, to divide the complexity of e-OSH into two stages by moving back to the two-stage hepatectomy strategy and resecting the primary at the stage of the resection of the lower liver volume.

**Figure 16:**
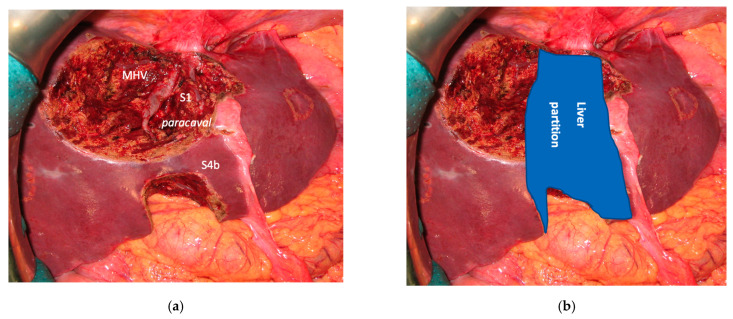
This is the first case we used the TWO-STAGE parenchyma-sparing resection, in 2020, to treat a left-sided colon cancer with 18 synchronous CRLM. At the first stage 60 mL of left liver were removed with resection of S4a partially extended to S8 with tangential resection of MHV and partial resection of S4b, left hemicolectomy was performed simultaneously. Two months after, 300 mL of right liver were removed in the second stage with the resection of S7 extended to the caudate processus an partially to S8 and S6 with complete exposure of RHV and metastasectomy of S5. Two months after the second stage a hepatic hilum recurrence occurred with infiltration of first-order GP. The MDT held a morbidity/mortality meeting and concluded that recurrence was due to a missed vanishing CRLM between MHV and hepatic hilum at the first stage. (**a**) Intraoperative field of the first stage showing MHV, the paracaval portion of S1 and the residual part of S4b. (**b**) With a little amount of additional liver volume (MHV + S1paracaval+ S4b) to complete the resection of S1 and S4 the vanishing CRLM would have to be removed. Abbreviations: CRLM: colorectal liver metastases; S: segment; MHV: middle hepatic vein; GP: glissonean pedicle; MDT: multidisciplinary team.

In 2020 we applied, for the first time, the two-stage parenchyma-sparing approach in a case of left-sided colon cancer with 18 bilobar synchronous metastases. At the first stage, left hemicolectomy was performed simultaneously with the minimal liver volume (60 mL) removal in the left liver (resection of S4a partially extended to S8 with tangential resection of MHV, partial resection of S4b and metastasectomy of S3), at histology the resection was R0 and TRG1. In the second stage, 300 mL of liver parenchyma were resected with a complex PSH in the right liver (resection of S7 extended to caudate processus and partially to S8 and S6 with complete exposure of RHV, metastasectomy of S5), at histology the resection was R0, TRG1 in all metastases but 1 of 6 mm which was TRG3. Two months after the second stage a hepatic hilum recurrence occurred with infiltration of the left biliary duct and portal vein. The patient was treated with chemotherapy and very complex R1 left hepatectomy with complex biliary and portal vein reconstruction, performed six months after the first stage. However, the disease recurred requiring percutaneous biliary drainage, the patient died for disease progression 2 years after the first surgical procedure. At the morbidity/mortality briefing our MDT concluded that the first recurrence at hepatic hilum was due to a vanishing CRLM at the hepatocaval confluence missed during the first stage. The MDT noticed that, at the first surgical stage, the vanishing CRLM at the hepatocaval confluence would have been removed by resecting a small additional amount of liver to complete the resection of S4 *en-bloc* with paracaval portion of S1, thus potentially avoiding disease recurrence (Figure 16).

As confirmed by this case, first-order GP division relapse is a highly challenging scenario as it may not be resectable and may hamper the administration of systemic treatments and strongly affect the patient’s quality of life due to biliary tree and portal vein involvement. To be sure to obtain R0 resection and/or remove disappeared CRLMs between MHV and the hilar plate we considered making completely free first-order GPs from liver parenchyma with a vessel-guided S4/S1 MLP instead of mini-mesohepatectomy [28] or liver tunnel [29]. We defined our mesohepatectomy “MLP” adding the terms “for liver partition” to emphasize the fact that S1 is included in the resection and to introduce the new concept of the liver as a paired organ (the “two livers” concept) in the management of CRLM.

Vessel-guided MLP of S4/S1 *en-bloc* with MHV is a minor resection (less than three adjacent liver segments) inducing a major hepatic scaffold modification transforming the liver in a paired organ as proposed by Bismuth et al. in 1989 [15,16]. Vessel-guided resection of S4/S1 *en-bloc* with MHV should be considered a complex core minor hepatectomy and an incidence of 13% of biliary leak is expected [30]. In our 15 consecutive vessel-guided MLP we have not observed any biliary leakage and we assume it might be due to the complete exposure of first-order GPs allowing precise biliostasis. On the other hand, MLP is associated with an increased risk of bleeding (80% of patients were transfused) which can be reduced, as reported in the literature [31]. MLP is a technically demanding procedure but may increase surgical opportunities offered by the improvement in patients’ life expectancy related to the new systemic armamentarium. One of these challenges is maintaining quality of life after a liver disease recurrence, up to 80% probability within 2 years of surgery in patients with a high tumor burden. In this regard, as mentioned above, first-order GP relapse should be avoided. To reduce this risk, we have reconsidered R0 surgery in the new era of R1 vascular liver resections [26]. Taking into account that vessel infiltration is not preventable [12], in selected cases of CRLM in contact with a single first-order GP, we propose R0 major resection of CRLMs *en-bloc* with the GP instead of a minor resection with R1-vascular detachment [26]. Obviously, in the case of contact with both first-order GPs, R1-vascular resection is mandatory and liver tunnel [29] with detachment is the surgery of choice. Thus, when CRLMs are at the hepatocaval confluence, close to but not in contact with first-order GPs, we prefer to spare liver parenchyma performing a MLP.

**Figure 17:**
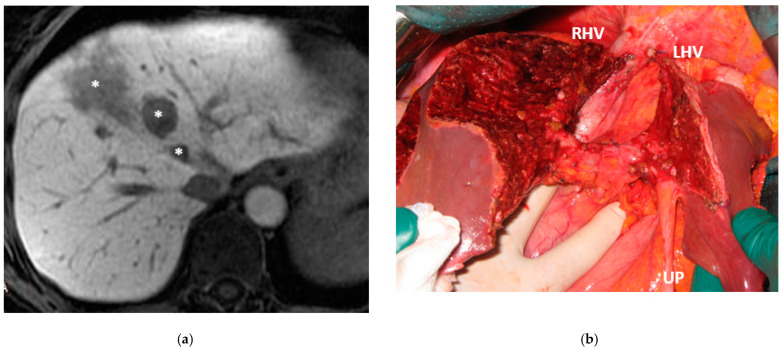
This case of major MLP of S1-S4-S8 with associated atypical resection of S3, S7 and S6, to treat 12 synchronous CRLMs, gives an example of location of CRLM at the hepatocaval confluence to better explicate the reasons behind the choice to perform the MLP. (**a**) Hepatobiliary phase of MR shows 3 CRLM at the hepatocaval confluence (marked with asterixis, *) which could be treated both with a liver tunnel or an MLP, or, of course, with a major extended resection. This patient had another 9 CRLMs, thus a high tumor burden associated with high probability of disease recurrence after surgery (up to 80% within the first 2 years). (**b**) The intraoperative field shows we chose to use MLP for four reasons: (1) to perform a parenchyma-sparing resection, not to prevent the opportunity of a subsequent re-resection; (2) to increase the chance to achieve an R0 resection; (3) to reduce the chance of recurrence around the first-order glissonean pedicles by removing all the tissue; (4) to prepare the liver for new application of liver augmentation techniques in a parenchyma-sparing context in the case of subsequent repeated liver resection. Abbreviations: *: CRLM; MLP: mesohepatectomy for liver partition; CRLM: colorectal liver metastases; S: segment; RHV: right hepatic vein; MHV: middle hepatic vein; UP: umbilical portion.

Certainly, CRLMs at the hepatocaval confluence can be technically treated with multiple different interventions ranging from extended right or left hepatectomies up to parenchyma-sparing liver resections such as the “liver tunnel” [29]. In most cases, preserving liver parenchyma is a proved advantage for the patient, but the technical ability and expertise of the surgeon cannot be underestimated since performing a parenchyma-sparing resection is much more difficult than a major extended liver resection. The aim of sparing liver parenchyma whenever possible, thus avoiding classical major hepatectomies, is a pillar of our clinical reasoning, including the option of performing a tumor detachment with R1-vascular resection. In selected cases we decided to use MLP, instead of liver tunnel, for the following reasons: (1) to perform a parenchyma-sparing resection, not to prevent the opportunity of a subsequent re-resection; (2) to increase the chance to achieve an R0 resection; (3) to reduce the chance of recurrence around the glissonean pedicles by removing all the tissue; (4) to prepare the liver for new liver augmentation techniques in a parenchyma-sparing context in the case of subsequent repeated liver resection. Figure 17 gives an example of location of CRLM at the hepatocaval confluence to better explicate the reasons behind the choice to perform the MLP.

We wanted to apply to GPs the same R0 parenchyma-sparing criterion used for HVs [12] and we have addressed the criticism of insufficient FLR volume by associating two contrasting paradigms, Torzilli’s parenchyma-sparing vessel-guided major hepatectomy [7] with Bismuth’s interpretation of the liver as a paired organ [16]. The role of MLP in liver regeneration is the same as a minor liver resection (less than three adjacent liver segments), since usually nearly 200 mL of liver parenchyma are resected. The main difference compared with all other parenchyma-sparing complex liver resections, maintaining the hepatic scaffold by preserving communicating veins between main hepatic veins, is that MLP results in a significant modification of the hepatic scaffold by resecting the middle hepatic vein with amputation of all communicating veins between right and left hepatic veins, obtaining two independent livers. As a consequence, the novel role of MLP in liver regeneration consists in the potential application of liver augmentation techniques in a parenchyma-sparing context when a subsequent liver resection is planned. In fact, MLP opens to new frontiers of parenchyma-sparing major liver resection such as PS-TSH associated to super-selective PVE when a single segment is preserved in the right liver or PS-ALPPS associated to portal vein ligation when segment four is preserved. This novelty has led us to explore an uncharged field of liver surgery and regeneration, planning interventions with impressive results in terms of liver regeneration.

Our new PS-TSH is defined as parenchyma sparing because it aims at preserving a single segment in the right liver instead of performing extended right hepatectomy (Figure 1). Super-selective PVE was performed for the first time in this setting in three patients with favorable short-term outcome. PS-TSH was really well tolerated, in particular considering the highest complexity of the second stage (i.e., in a 77-year-old patient we have preserved S6 with end-to-end RHV reconstruction, Figure 8) as it is performed in one of the two livers while the other remains untouched. For the same reason, eventually repeated liver resections are technically demanding due to adhesions, but still feasible (i.e., a patient was re-resected once in the left liver and twice in the right).

Taking into account that the liver can be considered a paired organ, we introduced the concept of e-ALPPS when first-order GP resection is planned and FLR volume is deemed insufficient. ALPPS is defined as enhanced because liver partition is obtained with two full thickness transection lines (MLP) instead of the single line as described in the classical ALPPS [6] with the advantage of leaving a well-vascularized liver parenchyma with no possibility for parenchymal debris. We have added to ALPPS the parenchyma-sparing concept, expanding the novelty introduced by Botea et al. [20] of preserving S4 whenever possible including the deportalized liver (Figure 9).

To date, in our institution, liver function evaluation with 99mTc-mebrofenin hepatobiliary scintigraphy is not available and liver volume is still the way to determine the adequate FLR [32]. In two cases of e-ALPPS (Figure 10, Figure 11, Figure 12 and Figure 13), adequate FLR was not achieved after the first stage despite MHV resection, which is considered a procedure accentuating FLR volume hypertrophy [33]. Our MDT, observing FLR volume increase after the resection of embolized liver in PS-TSH with SS-PVE, proposed the partial resection of deportalized liver as a boost for FLR regeneration up to the adequate volume. We observed for the first time a FLR volume increase beyond the classical ALPPS technique [6], which is considered the most powerful liver augmentation technique [34]. The regeneration observed is completely independent of the venous system and it is not possible to advocate the same mechanism of PVE, PVL or liver deprivation. The arterial system is most probably responsible for the observed volume increase and this is supported by Zhuo et al., who observed the same in a case of hepatic arterial infusion chemotherapy and arterial embolization in the deportalized liver with a huge HCC and inadequate FLR hypertrophy one month after stage 1 ALPPS [35].

The decision to perform an e-ALPPS instead of a TS-PSH with SS-PVE is based only on the necessity to resect first-order GP and not on the need to obtain faster liver regeneration. This is the first paper describing a new liver surgery based on the following paradigms: liver intended as a paired organ, R0 resection for maintaining patient’s quality of life after disease recurrence, vessel-guided major parenchyma-sparing liver resection and the use of liver augmentation techniques in a parenchyma preserving context. Performing very complex surgery in very complex patients needs highly dedicated MDT for the best estimation of the risk/expectations balance in each individual patient, including and valuing the expertise of dedicated oncologists, anesthesiologists, radiologists, pathologists and surgeons.

Overall, these demanding surgical procedures seem feasible since only one patient (who developed asymptomatic common trunk portal vein thrombosis after stage 1 e-ALPPS) died within 90 days after completing the liver augmentation strategy due to liver failure. Oncological outcomes are promising with a median RFS of 12 months and 1-year OS of 92% similar to other hepatic surgical series that mainly included patients with a lower liver disease burden requiring less complex surgery [27,36,37], though acknowledging the clear limitations of comparing different case series with highly different clinical scenarios. However, a longer follow-up and a larger sample size are needed to confirm these results.

In conclusion, vessel-guided MLP may have a role in the treatment of complex cases of synchronous CRLMs allowing to prevent disease recurrence at first-order GPs and thus maintaining quality of life in case of disease progression after surgery. Advanced surgical parenchyma-sparing techniques combined with liver augmentation techniques may offer new surgical strategies and therapeutic chances for patients with synchronous CRLMs to be properly placed in their *continuum of care*.

## 5. Conclusions

Surgical and oncological opportunities for patients affected by CRLMs have highly increased in the last decades, raising new questions about the best implementation of these options in daily clinical practice. The fine tuning of the therapeutic path for each individual patient is made possible only thanks to the common growth of a close MDT identifying the most clinically relevant goals in each case, properly estimating the cost/benefit balance of potential choices and finally choosing personalized approaches based on disease characteristics, their dynamic evolution across administered treatments and available techniques. 

Vessel-guided MLP has been developed at our institution with the objective to exploit the previous experience with parenchyma sparing and liver augmentation techniques to partially overcome the paradigm of minimal needed resection (that is, however, still a pillar of our philosophy of CRLM resection, especially when re-resections are on the horizon) to prevent predictable and immediately life-threatening relapses (i.e., those occurring at the first-order GPs). In our opinion, this may contribute to extend the reach of the feasibility of sound surgical procedures to improve our patients’ life expectancy.

## Data Availability

Data supporting reported results can be found at Azienda Ospedaliero-Universitaria Pisana, Pisa, Italy.
